# Braincase of *Siamraptor suwati* and insights into the cranial anatomy of Carcharodontosauria

**DOI:** 10.1371/journal.pone.0345155

**Published:** 2026-04-29

**Authors:** Soki Hattori, Duangsuda Chokchaloemwong, Soichiro Kawabe, Elena Cuesta, Masateru Shibata, Kazunori Miyata, Yoichi Azuma

**Affiliations:** 1 Institute of Dinosaur Research, Fukui Prefectural University, Eiheiji, Fukui, Japan; 2 Fukui Prefectural Dinosaur Museum, Katsuyama, Fukui, Japan; 3 School of Science Education, Faculty of Science and Technology, Nakhon Ratchasima Rajabhat University, Nakhon Ratchasima, Thailand; 4 Northeastern Research Institute of Petrified Wood and Mineral Resources, Nakhon Ratchasima Rajabhat University, Nakhon Ratchasima, Thailand; 5 SNSB-Bavarian State Collection for Paleontology and Geology, Munich, Germany; Soprintendenza Archeologia Belle Arti e Paesaggio Firenze Pistoia Prato, ITALY

## Abstract

Two partial theropod braincases, namely NRRU-F01020035 and F01020036, were recovered from the Lower Cretaceous Khok Kruat Formation in Nakhon Ratchasima, Thailand. Detailed observations on these two specimens revealed their carcharodontosaurian affinity based on the presence of several synapomorphies for the clade, such as partially-roofed anteromedial corner of the supratemporal fossa and a tall nuchal crest. In combination with the monospecific occurrence of Carcharodontosauria in the locality, both specimens can be attributed to *Siamraptor suwati*, although such braincase preserving the synapomorphic characters has been unknown in this taxon so far. In addition, the wedge-shaped frontoparietal suture and two deep pits on the lateral margin of the frontal shared in the two braincases, which have not been reported in any allosauroid, are shared by both specimens and thus can be regarded as additional autapomorphies of *Siamraptor*. The phylogenetic analysis incorporating information from the braincases confirmed the position of *Siamraptor* as an earliest-branching member of Carcharodontosauria as discussed in the original description, and further comparison with other allosauroid braincases revealed several aspects of the evolutionary history of carcharodontosaurian characters.

## Introduction

**Fig 1:**
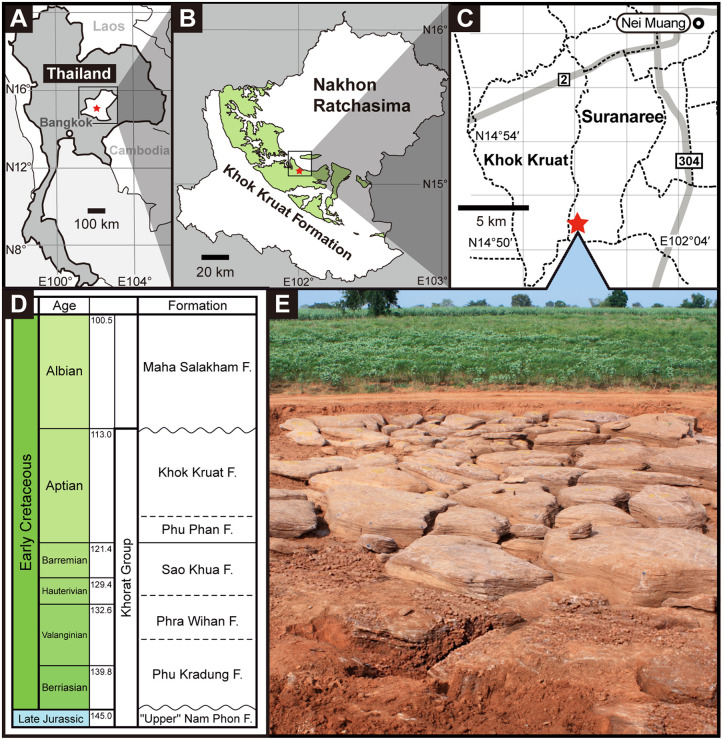
Locality map and stratigraphy of Khorat Group. A, map of Nakhon Ratchasima Province, Thailand; B, distribution map of the Khok Kruat Formation in Nakhon Ratchasima Province; C, enlarged locality map of Suranaree and Khok Kruat subdistricts with the subdistrict boundaries (broken lines); D, photograph of the excavation site; E, stratigraphic column of the Khorat Group (modified from [7]). Maps in A–C are adapted from Chokchaloemwong et al. [5] under CC BY 4.0. Red stars in A–C indicate the locality described in this study and the grey lines in C indicate national highways.

Since 2007, the Fukui Prefectural Dinosaur Museum (FPDM) and the Northeastern Research Institute of Petrified Wood and Mineral Resources, Nakhon Ratchasima Rajabhat University (NRRU) have conducted joint paleontological research in Thailand through the Japan-Thailand Dinosaur Project (JTDP) [1–5]. By 2013, 22 specimens of large-bodied theropods had been recovered from an outcrop of the Lower Cretaceous Khok Kruat Formation at Ban Saphan Hin (Ban meaning “village”), the Suranaree Subdistrict, northwest of the Mueang Nakhon Ratchasima District, Nakhon Ratchasima Province (Fig 1), and were subsequently identified as the earliest-diverging carcharodontosaur *Siamraptor suwati* [5]. Carcharodontosauria is a clade comprising large-bodied theropods flourished as apex predators in all land masses in the Late Jurassic to the Late Cretaceous period, except for Antarctica [6]. However, Asian carcharodontosaurs had been poorly known, especially on the early members of the clade. Therefore, the presence of *Siamraptor* bears an important role to understand the carcharodontosaurian evolutionary history.

Prior to 2007, a large theropod braincase, NRRU-F01020035, was recovered from an outcrop of the Khok Kruat Formation in Nakhon Ratchasima Province by Prasart Anankasa and his team. The precise locality of this specimen has long been uncertain for a long time due to a lack of accompanying field documentation. Recently, however, it was confirmed that NRRU-F01020035 had been collected between 2003 and 2005 at the Ban Saphan Hin locality, based on a collation of a diary by Pratueng Jintasakul, the museum’s director at the time, and a conversation with Prasart Anankasa.

Following the description of *Siamraptor* in 2019, a partial skull roof of a large theropod (NRRU-F01020036) collected in 2007 from the same locality was recognized in the collection. This specimen was recovered within a limited excavation area of approximately 300 m², together with several elements previously referred to *Siamraptor*, including right and left maxillae (NRRU-F1020004, F1020005), a middle caudal vertebra (NRRU-F1020017), and a manual ungual (NRRU-F1020018).

At the time of the original description of *Siamraptor*, only 22 skeletal remains referable to Allosauroidea had been recovered from the bone-bearing bed at the locality, and all were consistently identified as *Siamraptor*. For example, all of four partial mandibles recovered from this locality were referred to this taxon, despite substantial variation in their sizes [5]. The elements comprising NRRU-F01020035 overlap and share anatomical features with NRRU-F01020036, as well as other braincases of allosauroids. NRRU-F01020035 and F01020036 represent the only additional skeletal remains referable to Allosauroidea currently known and are provisionally referred to *Siamraptor*, pending for further confirmation.

Among allosauroids, almost complete braincases are known in both the basal allosauroids such as *Yangchuanosaurus* [8,9], *Sinraptor* [10–12] and *Allosaurus* [13–16] and the late-diverging carcharodontosaurs such as *Acrocanthosaurus* [17,18], *Tameryraptor* [19], *Carcharodontosaurus* [20–23], *Meraxes* [24], *Giganotosaurus* [25–27], *Shaochilong* [28], *Megaraptor* [29] and *Murusraptor* [30–32], although the latter three taxa have been sometimes considered as coelurosaurs rather than allosauroids [19,32,33]. In addition, a possible basal allosauroid *Asfaltovenator* also presents a well-preserved braincase [34]. Although there are such a handful of specimens of allosauroid braincases, information on those of basal carcharodontosaurs is limited. In this context, the probable braincase of the earliest-branching carcharodontosaur will provide important information to fill the gap in the evolutionary history of their braincase morphology.

### Geological setting

The Khok Kruat Formation is the uppermost unit of the Khorat Group and consists of meandering river deposit in the Khorat Basin, the southern part of the Khorat Plateau in northeastern Thailand [35]. The bone-bearing bed at the Ban Saphan Hin locality consists of lithofacies representing channel and bar deposits, characterized by medium- to coarse-grained sandstones and conglomerates with planar, cross, and large scaled epsilon-type cross laminations, and containing numerous clasts of clay rip-up pebble and rounded calcareous nodule granules [36]. The thickness of this bed is mostly 1–2 m and does not exceed 3 m.

Although the presence of carbonate clasts suggest that Khok Kruat Formation was deposited under the semi-arid climate [7], Amiot et al. [37] suggest that the Khorat Basin was formed under a subtropical or tropical climate and a semi-humid condition. The geological age of the Khok Kruat Formation has been dated to the Aptian based on the palynological data, occurrences of a freshwater hybodont *Thaiodus ruchae* and a basal ceratopsian *Psittacosaurus sattayaraki*, and the Albian–Cenomanian age inferred for the overlying Maha Sarakham Formation [7,38–41].

## Materials and methods

**Fig 2:**
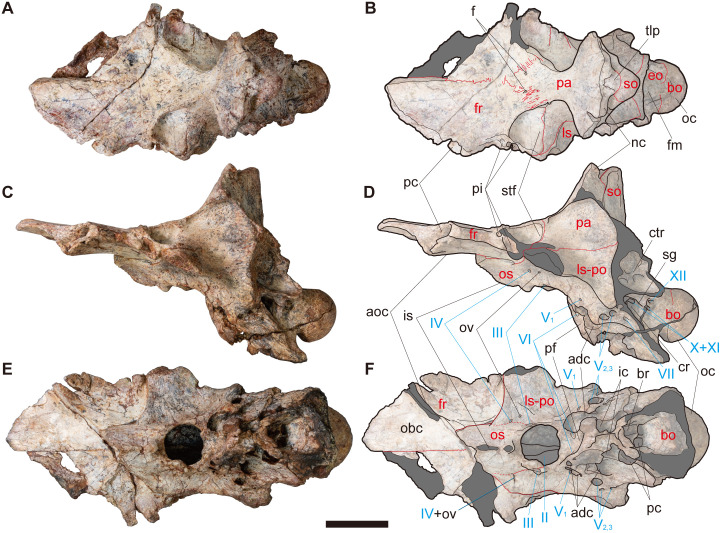
NRRU-F01020035 in dorsal (A, B), left lateral (C, D) and ventral (E, F) views. Red lines indicate sutures visible in the bone surface. Red and cyan characters indicate abbreviations of the skeletal elements and cranial nerves, respectively. Scale bar equals 50 mm. Abbreviations: adc, anterodorsal chamber of the rostral tympanic recess; aoc, anterolaterally oriented crest; bo, basioccipital; br, basisphenoid recess; cr, columeller recess; ctr, caudal tympanic recess; eo, exoccipital; f, foramen for some vein; fm, foramen magnum; fr, frontal; ic, internal carotid artery foramen; is, interorbital septum; ls-po, laterosphenoid-prootic complex; nc, nuchal crest; obc; olfactory bulbs contact; oc, occipital condyle; os, orbitosphenoid; ov, orbitocerebral vein; pa, parietal; pf, pituitary fossa; pc, prefrontal contact cavity; pi, pit; pc, posterior chamber for the caudal diverticulum of the rostral tympanic recess and the medial basioccipital diverticulum; sg, stapedial groove; so, supraoccipital; stf, supratemporal fossa; tlp, tongue-like process; II–XII, exits of each cranial nerve.

**Fig 3:**
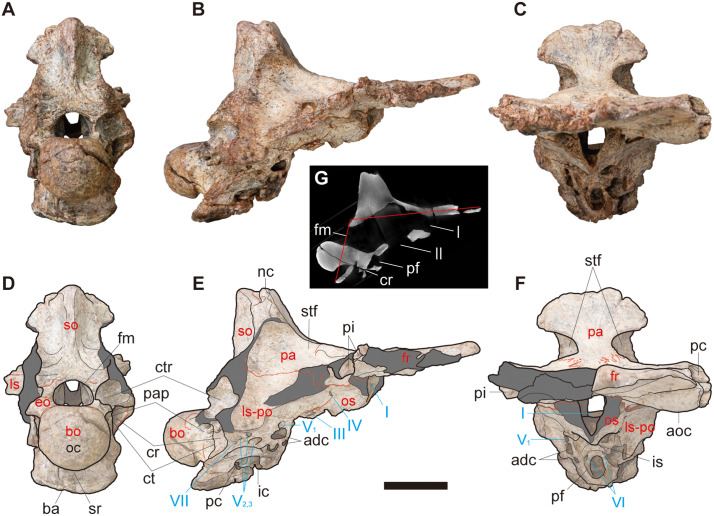
NRRU-F01020035 in posterior (A, D), right lateral (B, E) and anterior (C, F) views, and its sagittal section in right lateral view (G). Red lines indicate sutures visible in the bone surface in A–F and occipitofrontal angle in G. Red and cyan characters indicate abbreviations of the skeletal elements and cranial nerves, respectively. Scale bar equals 50 mm for A–F and 100 mm for G. Abbreviations: adc, anterodorsal chamber of rostral tympanic recess; aoc, anterolaterally oriented crest; ba, basioccipital apron; bo, basioccipital; cr, columellar recess; ct, crista tuberalis (metotic strut); cr, crack; ctr, caudal tympanic recess; eo, exoccipital; fm, foramen magnum; fr, frontal; ic, internal carotid artery foramen; is, interorbital septum; ls-po, laterosphenoid-prootic complex; nc, nuchal crest; oc, occipital condyle; os, orbitosphenoid; pa, parietal; pf, pituitary fossa; pc, prefrontal contact cavity; pap, paracondylar pocket; pc, posterior chamber for the caudal diverticulum of the rostral tympanic recess and the medial basioccipital diverticulum; pi, pit; so, supraoccipital; stf, supratemporal fossa; sr, subcondylar recess; I–VII, exits of respective cranial nerves.

**Fig 4:**
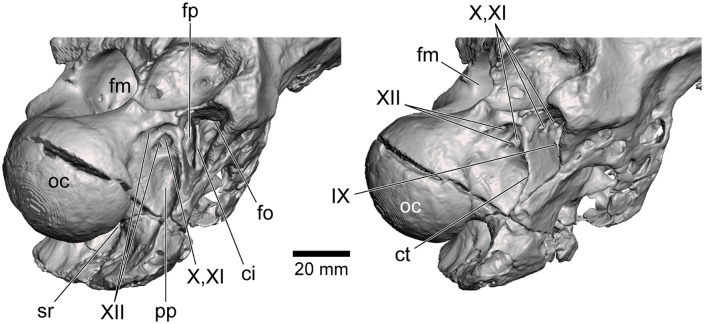
Exoccipital region in CT-based 3D rendered model of NRRU-F01020035 in posterolateral (left) and lateral slightly-posterior (right) views. Abbreviations: ci, crista interfenestralis; ct, crista tuberalis (metotic strut); fm, foramen magnum; fo, fenestra ovalis; fp, fenestra pseudorotunda; oc, occipital condyle; pp, paracondylar pneumatopore; sr, subcondylar recess; IX–XII, exits of respective cranial nerves.

**Fig 5:**
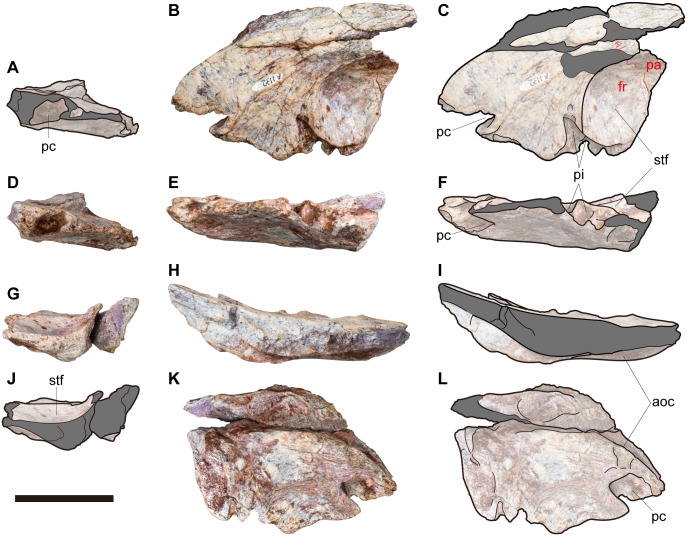
NRRU-F01020036 in anterior (A, D), dorsal (B, C), lateral (E, F), posterior (G, J), medial (H, I) and ventral (K, L) views. Red lines indicate sutures visible in the bone surface. Scale bar equals 50 mm. Abbreviations: alc, anterolaterally oriented crest; fr, frontal; pa, parietal; pc, prefrontal contact cavity; pi, pit; stf, supratemporal fossa.

An incomplete braincase (NRRU-F01020035) lacking at least both paroccipital processes and the cultriform process (Figs 2–4) and a partial skull roof (NRRU-F01020036) composed of an almost complete left frontal and a fragment of left parietal (Fig 5) are described in the present study. Both specimens are housed at a public and permanent repository in the collection of the Northeastern Research Institute of Petrified Wood and Mineral Resources, Nakhon Ratchasima Rajabhat University (NRRU), Thailand, and are accessible to all researchers. No permits were required for the described study, which complies with all the relevant regulations. The excavation and collection of fossil remains were agreed with the landowner and were officially reported to the Department of Mineral Resources, Thailand.

NRRU-F01020035 was analyzed using an industrial microfocus CT, TXS320- ACTIS (TESCO Co., Yokohama, Japan), at FPDM with the following parameters: voltage of 270 kV, current of 250 μA, and voxel size of 0.05 mm (x- and y-axes) and 0.05 mm (z-axis). Cranial endocast was reconstructed and segmented from the acquired CT images and exported as a polygon mesh to observe, measure and capture images for figures under the orthographic projection view using Amira (v 2020.1, Thermo Fisher Scientific; Waltham, USA).

To assess the phylogenetic position of *Siamraptor* incorporating new anatomical features recognized in the present study, they are included as a single operational taxonomic unit (OTU) and added to the recently published phylogenetic dataset [42], which is one of the updated versions of the dataset by Carrano et al. [43] that has been revised several times [30,44,45]. In addition, some scores of other OTUs are modified based on a review of the published literature as follows: character 100 in *Shaochilong* and *Giganotosaurus*, from 0 to 1; character 100 in *Carcharodontosaurus*, from? to 1; character 100 in *Acrocanthosaurus*, from 0 to 0&1; character 103 in *Murusraptor*, from 1 to 0; character 104 in *Carcharodontosaurus* and *Giganotosaurus*, from 1 to 0; character 104 in *Shaochilong*, from unknown to 0; character 104 in *Sinraptor*, from 0 to 1. For reasons of the modification, see “Implications for the braincase morphology of Allosauroidea” in Discussion.

The data matrices were managed using Mesquite 3.6 [46] and imported into TNT 1.5 [47] to perform a heuristic tree search and find the most parsimonious trees (MPTs). The heuristic tree search used the New Technology algorithms: sectorial searches, ratchet, tree-drifting, and tree fusing, using the default settings for all of them. These algorithms were applied to new searched trees using the driven search with a stabilization of the consensus twice with a factor of 25. Subsequently, the resulting most parsimonious trees (MPTs) were exposed to the branch-swapping algorithm of tree bisection reconnection (TBR). The characters were unordered. The MPTs found from both iterations were examined under the strict consensus, and the consistency index (CI) and retention index (RI) were obtained using the “stats.run” script of TNT. The branch support was tested using the methodology proposed by Goloboff et al. [48] to calculate Bremer Support values, retaining up to 99,999 suboptimal trees that are up to 5 steps longer than the MPTs.

### Systematic paleontology

Dinosauria Owen, 1842 [49].

Theropoda Marsh, 1881 [50].

Tetanurae Gauthier, 1986 [51].

Allosauroidea Marsh, 1878 [52].

Carcharodontosauria Benson, Carrano and Brusatte, 2010 [53].

*Siamraptor* Chokchaloemwong, Hattori, Cuesta, Jintasakul, Shibata and Azuma, 2019 [5].

Type species: *Siamraptor suwati* Chokchaloemwong, Hattori, Cuesta, Jintasakul, Shibata and Azuma, 2019 [5].

### Emended diagnosis

Allosauroid theropod with following characters autapomorphic among allosauroids: jugal with straight ventral margin, and dorsoventrally deep anterior process below the orbit; frontoparietal suture directed posterolaterally on the skull table; two deep pits on the lateral margin of the anterolateral corner of the supratemporal fossa of the frontal; surangular with a deep oval concavity at the posterior end of the lateral shelf and four posterior surangular foramina; long and narrow groove along the suture between surangular and prearticular; articular with a foramen at the notch of the suture with prearticular; anterior cervical vertebra with an additional pneumatic foramen excavating parapophysis; cervical and posterior dorsal vertebrae penetrated by a pair of small foramina bilaterally at the base of each neural spine.

### Referred specimens

An incomplete braincase lacking at least both paroccipital processes and the cultriform process (NRRU-F01020035) and a partial skull roof composed of an almost complete left frontal and a fragment of left parietal (NRRU-F01020036).

### Locality and horizon

The Lower Cretaceous (Aptian) Khok Kruat Formation of the Khorat Group cropping out in Ban Saphan Hin, Suranaree Subdistrict, Mueang Nakhon Ratchasima District, Nakhon Ratchasima Province, Thailand.

NRRU-F01020035 was analyzed using an industrial microfocus CT, TXS320-ACTIS (TESCO Co., Yokohama, Japan), at FPDM with the following parameters: voltage of 270 kV, current of 250 µA, and voxel size of 0.05 mm (x- and y-axes) and 0.05 mm (z-axis). Cranial endocast was reconstructed and segmented from the acquired CT images and exported as a polygon mesh to observe, measure and capture images for figures under the orthographic projection view using Amira (v 2020.1, Thermo Fisher Scientific; Waltham, USA).

### Description

#### Frontal.

In NRRU-F01020035, both frontals are present as wedge-shaped plates as in other allosauroids, although the anterior and lateral margins are damaged (Figs 2, 3). In NRRU-F01020036, although the left frontal lacks its anteromedial and posterolateral parts, the anterolateral margin is better preserved than those of NRRU-F01020035 (Fig 5). The size and morphological features of the frontal are quite similar between these two specimens.

In comparison with other allosauroids, the frontal is lateromedially broad even in the anterior part (Fig 2A, B) as in *Sinraptor hepingensis* [12] and carcharodontosaurids [17,19,21,25,54] but unlike *Allosaurus* and *Sinraptor dongi*, in which the anterior part is much narrower than the posterior part in dorsal view [10,13,15]. The bone gradually tapers anteriorly in dorsal view to form a blunt but pointed end at its anteromedial margin. The dorsal surface of the frontal except for the supratemporal fossa forms an anterior part of the skull table as a flat plane like those of other allosauroids [10,12,13,15,17,19,21,25] but unlike the narrow one of *Murusraptor* [31] and the sagittal crest of *Shaochilong* [28]. The interfrontal suture is straight in dorsal and ventral views in its anterior two-thirds but becomes intricately sculptured in the posterior one-third. Both frontals are connected even at their anterior ends, unlike most allosauroids, in which both frontals diverge before reaching their anterior ends [10,13,30]. At the posterior end of the interfrontal suture, the frontoparietal suture emerges as a deeply interdigitating line that is directed posterolaterally on the skull table forming a slight dorsal swelling. Such direction of the frontoparietal suture accompanied with the wedge-shaped anterior margin of the parietals is unknown in other allosauroids, in which the suture is directed laterally even on the skull table [10,12,13,15,30,54]. There is a pair of small foramina possibly for the exits of some vascular components at the middle of the frontoparietal suture on the skull table (Fig 2B). The frontoparietal suture enters into the supratemporal fossa with a slight lateral swelling (frontoparietal process *sensu* [54]) that is less distinctive than those of *Sinraptor* [10] and *Eocarcharia* [54], and becomes a straight line running laterally on the bottom of the fossa as in other allosauroids. The frontoparietal suture is located more anteriorly on the skull table than in the supratemporal fossa as in *Sinraptor* (Fig 4 in [12]; Fig 7 in [10]) but unlike *Allosaurus*, in which the one is located more posteriorly on the skull table than in the supratemporal fossa (Fig 11 in [13]; Fig 2 in [15]). At the anteromedial corner of the supratemporal fossa, the dorsal rim of the fossa slightly protrudes posterolaterally to partially roof the fossa (Figs 2A, B, 5B, C) as in some carcharodontosaurids [43,54]. At the lateral end of this rim, the lateral margin of the bone is damaged but preserves two deep pits excavating the frontal medially (Figs 2A–D, 3B, E, 5B, C, E, F). These pits probably related to the postorbital contact and a similar excavation is also seen in *Eocarcharia* [54] whereas the surface is rather flat or convex in other allosauroids [14,17,25,31,55]. These pits are separated by a thick lamina into the anterodorsal and posteroventral ones unlike an almost single slot of *Eocarcharia* [54]. In NRRU-F01020035, the ventral rim of the posteroventral pit protrudes somewhat ventrally from the remainder of the ventral surface of the bone, whereas this protrusion is broken in NRRU-F01020036.

On the ventral surface, there is an anterolaterally oriented crest aligned with the broken anterior margin of the orbitosphenoid (Figs 2E–F, 5K, L). At the lateral end of the crest, the lateral margin of the frontal bears a deep pit penetrating the frontal posteromedially to receive the posterior apex of the prefrontal as in *Allosaurus* [13] and *Carcharodontosaurus* [21]. Anteromedial to the crest, the thickness of the frontal becomes about half of the posterolateral part of the bone and exhibits a shallowly-concave surface representing the area of the contact for the olfactory bulbs (Fig 2E, F). Posterior to the crest, the medial and posterior margins of the ventral surface of the frontal are covered by the orbitosphenoid and the laterosphenoid, respectively.

#### Parietal.

In NRRU-F01020035, both parietals are well preserved but lacking a large part of the transverse nuchal crest (Figs 2, 3). In NRRU-F01020036, only the anteriormost part of the left parietal adhered to the frontal is preserved (Fig 5). In contrast to the frontals, the interparietal suture is obliterated (Fig 2A, B) so that both parietals are completely fused as in carcharodontosaurids [17,19,21,25,28] but unlike basal allosauroids [10,13,15] and *Murusraptor* [30]. In dorsal view, the parietals extend anteriorly to form a wedge invading the interfrontal suture and laterally to meet the suture against laterosphenoids. In lateral view, the parietal-laterosphenoid suture is oriented almost horizontally (Figs 2C, D, 3B, E).

In dorsal view, the parietals are largely occupied by the supratemporal fossae. The dorsal surface between supratemporal fossae forms a supratemporal crest with a flat plane as a posterior part of the skull table (Figs 2A, B, 5B, C). The lateral width of this crest is similar to that of *Allosaurus* (Plate 2 in [13]), which is narrower than those of *Acrocanthosaurus*, *Carcharodontosaurus* and *Giganotosaurus* [17] but wider than those of *Shaochilong* [28] and *Murusraptor* [30]. Posteriorly, the parietals are projected dorsally with the skull table gradually becomes facing anterodorsally and spread laterally to form a prominent dorsal margin of the nuchal crest (Figs 2A–D, 3B, C, E, F). In anterior view, the center of the dorsal margin is concave as in *Allosaurus* (Fig 13 in [13]) but unlike the prominent top of the crest seen in *Sinraptor* [10] and *Giganotosaurus* [25]. The dorsal margin of the nuchal crest does not extend much above the supraoccipital (Figs 2C, D, 3B, E) as in *Sinraptor* [10] and is tall as in some abelisauroids and carcharodontosaurs [43]. In dorsal view, there is a small tongue-like process on the midline partially overlapping the dorsal surface of the supraoccipital as in *Sinraptor* [10] and *Allosaurus* [15] but is much smaller than that of *Giganotosaurus* [25].

#### Remainder of the braincase.

The remainder of the braincase is represented only by NRRU-F01020035 lacking at least opisthotics and the parasphenoid. The foramen magnum is circular, which is 3 cm wide and 2 cm high in posterior view (Fig 3A, D). The occipital condyle is 5 cm wide and 4 cm high in posterior view. In lateral view, the angle between the skull roof and the occiput (occipitofrontal angle) is obtuse (Fig 3G) as in other allosauroids except for *Allosaurus* and *Acrocanthosaurus* [25,31,43].

The orbitosphenoids are the anteriormost elements of the braincase except for the frontals (Figs 2C–F, 3B, E). The orbitosphenoids are completely fused with each other to form a low median ridge, which probably correspond to the interorbital septum seen in *Acrocanthosaurus* [17], *Giganotosaurus* and *Carcharodontosaurus* [25]. The anterior part of the orbitosphenoid contacts the frontal, as in carcharodontosaurids [17,21,28,54], but unlike *Sinraptor* and *Allosaurus*, in which the one is bordered only by the laterosphenoid [10,14,56]. Although the anterior part of the orbitosphenoid may pertain to the sphenethmoid, as suggested for *Carcharodontosaurus iguidensis* [21] and possibly for *Acrocanthosaurus* [57], there is no obvious evidence of its ossification in NRRU-F1020035. In ventral view, the anterior margin of the left orbitosphenoid is aligned with the anterolaterally oriented crest (Figs 3E, F), as in *Sinraptor* (Fig 7E in [10]) and *Allosaurus* (FPDM-V-9672). However, this margin is somewhat broken in both orbitosphenoids along the same straight line. Therefore, the orbitosphenoid might extend slightly more anteriorly as in *Acrocanthosaurus* (Fig 16 in [17]). A sizable opening for the exit of the cranial nerve (CN) I (olfactory nerve) excavates the medial parts of the orbitosphenoids anteroposteriorly. The mesethmoid is absent in this specimen, resulting in a single opening for CN I, as in *Sinraptor* [10] and multiple specimens of *Allosaurus* [13,16,17].

Posterior to the orbitosphenoids, there is a large circular opening corresponds to the exit of CN II (optic nerve) with the path of the pituitary (Fig 3F). The flange of bone overlying and separating them in other theropods (e.g., [17,25,58]) is absent. It is uncertain whether the flange was not ossified or eroded by the weathering. Such a large opening is similar to the exit of CN II of *Allosaurus* [14]. However, the opening of NRRU-F01020035 is only anteriorly bordered by the orbitosphenoids, whereas is completely surrounded by the orbitosphenoids in *Allosaurus* [14]. The exit of CN III (oculomotor nerve) lies adjacent to the lateral margin of the exit of CN II and, being bordered anteromedially by the orbitosphenoid and posterolaterally by the laterosphenoid, marks the approximate posterior end of the suture between these elements.

The laterosphenoid is covered dorsally by the frontal and the parietal (Figs 2D, 3E) as in other allosauroids. While the laterosphenoid-prootic suture is not obvious, the probable remnant of the laterosphenoid-orbitosphenoid suture is partially visible. The laterosphenoid has a barlike lateral extension but its distal end, namely the distal cotylus that contacts the postorbital, is broken (Fig 2F). The anteromedial part of the left laterosphenoid has a pair of small foramina. The more anteromedial one corresponds to the exit of CN IV (trochlear nerve) because it is present on the remnant of the orbitosphenoid-laterosphenoid suture along with the exit of CN III as in most theropods [14,27,59]. The other, more posterolateral one probably corresponds to the orbitocerebral vein. These foramina seem to be merged in the right laterosphenoid as in *Murusraptor* [31] because there is only a single, slightly larger foramen that intercepts the remnant of the orbitosphenoid-laterosphenoid suture.

The pituitary (hypophyseal) fossa is situated posteroventral to the exit of CN II and excavates the braincase posteriorly (Fig 2D, F). This fossa is possibly composed of the complex of basisphenoid and parasphenoid, but there is no obvious suture line to distinguish them. Left and right exits of CN VI (abducens nerves) are visible as a pair of small foramina within the anterior part of the fossa as in *Shaochilong* [28], *Carcharodontosaurus* [23] and *Giganotosaurus* [25,26] unlike those on the outside of the pituitary fossa and separated by a median ridge seen in *Acrocanthosaurus* [18] and *Shaochilong* [31]. The deepest part of the pituitary fossa has a pair of exits that correspond to the internal carotid artery foramina connected to the rostral (anterior) tympanic recess as in *Shaochilong* [28].

The prootic is completely fused with the laterosphenoid as the suture between them is not visible (Figs 2, 3). The posteroventral region of this laterosphenoid-prootic complex is perforated by exits of CNs V and VII (trigeminal and facial nerves). There are four trigeminal foramina (exits of CN V) penetrating medially into the braincase on the right side while only three on the left side (Fig 2D, F). Among the trigeminal foramina, the anterior one is separated from the posterior foramina as in *Allosaurus* [14,16] and *Murusraptor* [30], despite the posterior foramina are merged into one in the latter two taxa. Therefore, the area anterior to the posterior foramina is probably occupied by the laterosphenoid as in these two taxa [14,16,31]. The anterior foramen is the exit of CN V_1_ (ophthalmic branch of CN V), which is situated posterolateral to the exits of CNs II and III and penetrates posteromedially into the braincase. Somewhat posterior to the exit of CN V_1_, there are three openings for CN V_2,3_ (maxillary and mandibular branches of CN V) on the right side, whereas only two are on the left side. Based on their position, the two smaller openings on the right correspond to the smaller opening on the left because both are dorsal to the largest opening (Figs 2D, 3E). Each of these two sections possibly corresponds to each branch but cannot be determined here. Posterior to these trigeminal foramina, there is a small opening for the exit of CN VII penetrating the prootic region. When the frontal is horizontal, the exit of CN V_1_ is located ventral to the nuchal crest, whereas those of CNs V_2,3_ and VII are located more posteriorly. Posterodorsal to the exit of CN VII, there is a large foramen that corresponds to the columellar recess [56]. Ventral to the exit of CN VII, the rostral tympanic recess excavates the braincase anteromedially, which seems to be covered laterally by the basisphenoid if preserved. The anterodorsal end of the rostral tympanic recess is connected to an oval chamber excavating the region ventromedial to the exit of CN V_1_. This chamber is almost completely exposed on the left side due to the absence of its lateral wall, which is partially preserved in the right side. Ventral to the entrance to this anterodorsal chamber is the internal carotid artery foramen as a junction to the pituitary fossa. Posterior to this foramen, there is an entrance for an internal chamber invading the braincase toward the neck of the occipital condyle (see “Endocranial cavities” for details).

**Fig 6:**
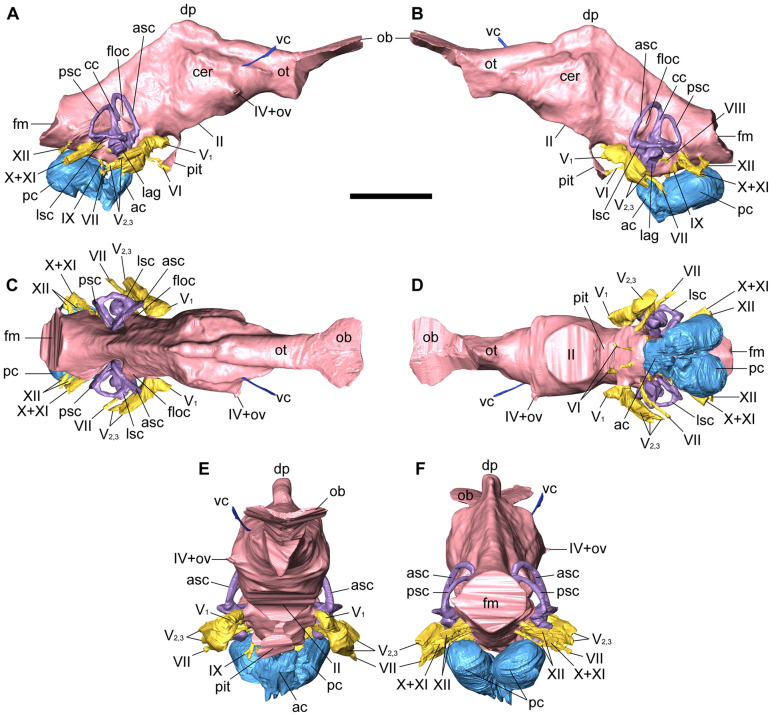
Cranial endocast of NRRU-F01020035 in right lateral (A), left lateral (B), dorsal (C), ventral (D), anterior (E), and posterior (F) views. Scale bar equals 100 mm. Abbreviations: ac, subsidiary diverticulum occupying the anterior chamber; asc, anterior semicircular canal; cc, common crus; cer, cerebral hemisphere; dp, dural peak; floc, floccular recess; fm, foramen magnum; lag, lagena; lsc, lateral semicircular canal; ob, olfactory bulb; ot, olfactory tract; ov, orbitocerebral vein; pit, pituitary; pc, complex of the caudal diverticulum of the rostral tympanic recess and the medial basioccipital diverticulum occupying the posterior chamber; psc, posterior semicircular canal; vc, venous canal; II–XII, cranial nerves.

**Fig 7:**
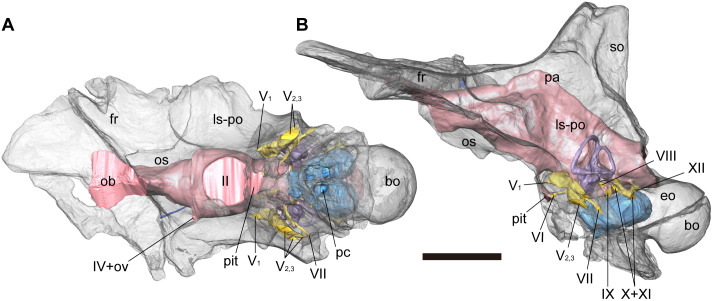
Cranial endocast with transparent braincase of NRRU-F01020035 in ventral (A) and left lateral (B) views. Scale bar equals 100 mm. Abbreviations: bo, basioccipital; eo, exoccipital; fr, frontal; ls-po, laterosphenoid-prootic complex; ob, olfactory bulb; os, orbitosphenoid; ov, orbitocerebral vein; pa, parietal; pc, complex of the caudal diverticulum of the rostral tympanic recess and the medial basioccipital diverticulum occupying the posterior chamber; pit, pituitary; so, supraoccipital; II-XII, cranial nerves.

Below the laterosphenoid- prootic region, the basisphenoid and parasphenoid are not preserved or are extremely poorly preserved (Figs 2D, 3E). Although the pituitary fossa could be interpreted as a remnant of the basisphenoid, this interpretation is uncertain, and the posterior part of the fossa may instead be formed by the laterosphenoid, as in *Murusraptor* (Fig S2 of [31]). The dorsal part of the basisphenoid recess is formed by the basioccipital as a large opening directed posteroventrally anterior to the broken basal tubera (Fig 2F). The basisphenoid recess is divided anteroposteriorly and the posterior part is further divided transversely by thin septa. The anterior part is a fossa with a faint connection to an internal chamber invading the braincase anterodorsally, toward the region just posterior to the pituitary fossa (Figs 6, 7; see “Endocranial cavities” for details). However, a large crack just across this connection indicates that the wall separating the chamber from the fossa has been lost by fracture (Fig 3G). The posterior parts are connected to the two larger chambers mentioned above, which communicate with the rostral tympanic recesses and extend into the neck of the occipital condyle.

The posterodorsal region of the braincase is composed of the supraoccipital, which contacts the parietal anteriorly and the exoccipital-opisthotic complexes lateroventrally (Figs 2B, D, 3D, E). The lateral parts of the supraoccipital are broken. In dorsal view, the suture line between the supraoccipital and the parietal is invaded by the tongue-like process of the parietal (Fig 2B) as in other allosauroids [10,15,25]. There is a dorsal projection of the supraoccipital (supraoccipital knob) along with the median ridge on the posterior surface (supraoccipital crest) as in other allosauroids [10,21,24,25,28] except for *Acrocanthosaurus* and some specimens of *Allosaurus*, in which the knob is divided into two by a midline fold [17]. The width of the supraoccipital knob (39.60 mm) is larger than that of the foramen magnum (29.93 mm) as in other allosauroids [43]. Although the suture line against the exoccipitals is indistinct near the dorsal margin of the foramen magnum, the remaining part of the suture indicates that the contribution of the supraoccipital to the foramen magnum is very narrow or absent (Fig 3D).

In posterior view, the exoccipitals form lateral edges of the foramen magnum (Fig 3D). Whether or not the exoccipitals are separated by the supraoccipital on the dorsal edge of the foramen magnum is ambiguous because the suture against supraoccipital is invisible in that part. In contrast, the exoccipitals are clearly separated by the basioccipital on the ventral edge of the foramen magnum. At the midheight of the foramen magnum, a horizontal suture-like line is present as in *Murusraptor* (Fig 6E’ in [30]). In dorsal view, sutures against the basioccipital are present just anterodorsal to the condylar surface (Fig 2B), thus exoccipitals have only a little contribution to the occipital condyle unlike *Allosaurus*, in which the exoccipitals occupy a conspicuous part of the condyle [13]. Dorsolateral to this suture, a large pneumatic cavity probably correspond to the caudal tympanic recess invades the base of the paroccipital process (Fig 3D, E) as in *Sinraptor* and many other theropods [56].

The basioccipital composes most of the occipital condyle and makes a small contribution to the floor of the foramen magnum (Fig 2B). Just below the occipital condyle, the basioccipital forms a broad, plate-like surface (“basioccipital apron” sensu [60,61]) that faces posterodorsally and is slightly wider than the occipital condyle in posterior view (Fig 3D). The basioccipital apron continues posteroventrally with forming a rough and concave posterodorsal surface toward the basal tubera, whereas the tuber itself is not preserved in this specimen. The concave posterodorsal surface becomes a deep fossa at the dorsal margin of the basioccipital apron excavating the base of the neck of occipital condyle anterodorsally (Fig 4A). This fossa probably corresponds to the subcondylar recess but is not connected with any internal pneumatic cavities unlike those of *Shaochilong* [28] and *Murusraptor* [30]. The lateral width of this fossa (21 mm) is much narrower than that of the occipital condyle (51 mm) as in most theropods [43].

Anterolateral to the occipital condyle and posteroventral to the caudal tympanic recess, there is a large fossa correspond to the paracondylar pocket [28,62] or paracondylar recess ([56,58,63]; Fig 3D, E). The dorsal part of this fossa bears two foramina separated dorsoventrally by a septum. The dorsal foramen represents the exit of the posterior branch of CN XII (hypoglossal nerve). The ventral foramen is further separated mediolaterally by a vertical septum (Fig 4A) as in *Giganotosaurus* [27,64]. The medial, smaller one corresponds to the anterior branch of CN XII, whereas the lateral, larger foramen is interpreted as the vagal foramen, representing the caudal opening of a subdivided metotic fissure and transmitting CNs X and XI (vagus and accessory nerves; Fig 2D). In total, there are three foramina as in *Giganotosaurus* [18], *Shaochilong* [28] and *Murusraptor* [31] on the right side, unlike *Sinraptor* and *Allosaurus*, in which there are only two foramina because CN XII exits only through one foramen [16,56]. The remaining ventral part of the paracondylar pocket probably corresponds to the paracondylar pneumatopore, although which is more deeply excavated in carcharodontosaurids to lead into an extensive recess below the endocranial cavity [21,25,28,43]. The anterior margins of these foramina and a fossa is formed by a thin bony web as a remnant of the crista tuberalis (metotic strut). Anterior to this bony web, there is a large triangular cavity representing the columellar recess excavating the braincase anteromedially.

The dorsal and anteroventral rims of the columellar recess are formed by a thin ventral wall of the caudal tympanic recess and a thicker lamina, respectively (Figs 2D, 3D, E). The columellar recess accomodates two foramina separated by a vertical septum, namely the crista interfenestralis (Fig 4). The anterolateral division is the fenestra ovalis (or vestibularis) excavating the braincase anterodorsally to connect with the cochlear duct. The posteromedial division is the fenestra pseudorotunda (or fenestra cochleae). The anterior end of the dorsomedial corner is the deepest part that is excavated by an opening for CN VIII (vestibulocochlear nerve). Posteriorly, the dorsomedial corner has two additional openings of the vagal canal transmitting CNs X and XI in the right side, while there is only one opening in the left side. The area ventral to them is excavated by an opening for CN IX (glossopharyngeal nerve).

#### Cranial endocast.

The digital cranial endocast reconstructed from the well-preserved braincase (NRRU-F01020035) is nearly complete and consists of the cavities for the brain and inner ears, canals for cranial nerves and blood vessels, and pneumatic cavities (Figs 6, 7). The endocast preserves the olfactory bulbs and cerebral hemispheres, as well as the midbrain, hindbrain, and pituitary. Some dural sinuses are visible on the endocast, but cranial nerves and blood vessels themselves cannot be observed in the CT slices and were therefore not reconstructed. The endocast measures 175 mm long from the anterior end of the olfactory bulbs to the foramen magnum and has a maximum width of 42 mm at the lateral processes of the cerebral hemispheres. The volume of the endocast is approximately 124 ml, excluding canals for cranial nerves and blood vessels.

Overall, the cranial endocast is dorsoventrally low and mediolaterally narrow, with bulges indicative of forebrain, midbrain, and hindbrain structures. The cephalic flexure (angle between the forebrain and the midbrain) and the pontine flexure (angle between the midbrain and hindbrain) are at 130 and 150 degrees, respectively, which makes the forebrain slightly upward to the hindbrain, but the two are approximately parallel. The angles of the cephalic and pontine flexures are similar to those of *Sinraptor* [56], *Tameryraptor* [19], *Carcharodontosaurus* [23] and *Giganotosaurus* [25,26]. This makes the endocast elongate anteroposteriorly, unlike those of *Allosaurus*, *Ceratosaurus*, *Majungasaurus* and *Viavenator*, which are dorsoventrally high [16,65–67]. There are no traces of sutures between the bones of the braincase on the surface of the endocast, confirming that the braincase belongs to a subadult or adult specimen.

#### Forebrain.

The anteriormost portion of the cranial endocast represents the olfactory bulbs and tract. Only the dorsal margin of the olfactory bulbs is visible as impressions on the ventral surface of the frontal as in other theropods [16]. Due to the absence of bony septum, the olfactory bulbs are reconstructed as a single structure expanded anterolaterally, but in dorsal view, it shows a heart shape, with its midline slightly depressed along the antroposterior axis. The olfactory tract is triangular in its cross-section with a flat dorsal surface and relatively short in comparison to these of *Carcharodontosaurus* [23] and *Giganotosaurus* [25,26]. The ventral apex of this tract becomes rounded posteriorly to merge with the cerebral hemispheres, while the flat dorsal margin continues posteriorly to the approximate midpoint of the cerebral hemispheres. The lateral process on the anterior part of the cerebral hemisphere marks the part of the endocast with the greatest mediolateral width. The venous canal, reconstructed on the right side only, extends anterolaterally from the anterior dorsolateral margin of the cerebral hemisphere as in *Majungasaurus* [58] and *Allosaurus* [16,66]. Ventral to the cerebral hemisphere is the large circular exit of CN II (optic nerve) with the path of the pituitary (Fig 7A). The dorsalmost part of the endocast, the dural peak, is at the same anteroposterior level as the caudal margins of the CN II exit and the cerebral hemispheres.

#### Midbrain.

There is no obvious representation of the optic lobes (optic tecta) and CNs III (oculomotor nerve) and IV (trochlear nerve). The midbrain is inclined posteroventrally along with the occiput (occipital plate) as in *Carcharodontosaurus* [23] and *Giganotosaurus* [25,26]. Posterior to the cerebrum, the dorsal surface of the endocast above the hindbrain is marked by a pronounced ridge that continues caudally from the dural peak.

#### Hindbrain.

Boundaries between the cerebellum, medulla, and pons of the hindbrain are not clearly defined in the cranial endocast. However, there is a distinct floccular process on each side (Figs 6, 7C), which in life housed the floccular lobe of the cerebellum, as in other theropods [10,27,66,68,69]. The floccular process extends posterolaterally to enter the region surrounded by the anterior semicircular canal. The floccular process is elongated dorsoventrally and slopes anteroventrally along the long axis of the anterior semicircular canal. The subtle swelling dorsal to the floccular process probably corresponds to a vascular component (Fig 6A, B) such as the vena capitis dorsalis of *Carcharodontosaurus* [23] and the caudal middle cerebral vein of *Murusraptor* [31] and *Giganotosaurus* [27]. Anteroventral to the floccular process, CN V (trigeminal nerve) is represented by a single root on the lateroventral margin of the hindbrain as in other allosauroids [16,18,23,27,31,56]. CN V extends anteroposteriorly to form an anterior exit for CN V_1_ (ophthalmic division) and two or three posterior exits for CN V_2,3_ (maxillary and mandibular divisions). An enlarged region at the base of these divisions indicates the location of the trigeminal ganglion [16,31]. A similar pattern, in which the ophthalmic, maxillary and mandibular branches diverge from a shared exit region, is also common in other tetanurans (e.g., *Allosaurus*, *Struthiomimus*, *Dromaeosaurus*, *Troodon*), although tyrannosaurids have ophthalmic and maxillomandibular canals that branch separately from the endocranial cavity [67]. CN VI (abducens nerve) projects anteroventrally from the ventral part of the hindbrain and enters into the pituitary fossa. CN VII (facial nerve) emerges posterior to the exits of CN V_2,3_ and extends lateroventrally. CN VIII (vestibulocochlear nerve) emerges ventral to the endosseous labyrinth and anterodorsal to the passage of CN IX (glossopharyngeal nerve). CN IX has its own passage completely separated from those of CNs X and XI (vagus and accessory nerves) as in *Carcharodontosaurus* [23], unlike that of *Allosaurus* conjoined only with CN X [16] nor with CNs X and XI as in *Acrocanthosaurus* [18], *Giganotosaurus* [27] and *Murusraptor* [31]. CNs X and XI shares the passage as the vagal canal directed posterolaterally, although its middle part is bifurcated in the left side. The endocast has a distinct lateral process dorsomedial to the metotic fissure. There are two passages for CN XII (hypoglossal nerve), which arise from different roots and extend posterolaterally from the posteroventral aspect of the hindbrain.

#### Diverticulum.

In the cranial endocast, three pneumatic chambers for the diverticula located in the basicranium ventral to the hindbrain are reconstructed (Figs 6, 7). These are the anterior one and the posterior two chambers.

The anterior chamber extends anterodorsally and expands anterolaterally. There are sagittal grooves on the dorsal and anteroventral surfaces, indicating a subtle division of the anterior chamber into left and right. Although the anterior chamber has a small opening in the anterior part of the basisphenoid recess, a large crack just across this opening indicates that the wall separating two chambers has been lost by fracture (Fig 3G). Instead of the external opening, the anterior chamber has a broad communication with the posterior chambers. Therefore, the anterior chamber probably housed the subsidiary diverticulum derived from the one occupying the posterior chamber.

The posterior chambers are posteriorly elongated and posterolaterally dilated. The relative position of the posterior chamber is similar to that of the caudal diverticulum of the rostral tympanic recess (cRTr) and the medial basioccipital diverticulum (MBd) in other avetheropods [70]. In addition, the posterior chamber communicates with the posteroventral part of the rostral tympanic recess as in cRTr, and with the dorsal apex of the basisphenoid recess as in MBd (Figs 2F, 7A). Each posterior chamber is elongated posteriorly as in cRTr [70]*.* However, the posterior chamber of *Siamraptor* is oval and expanded posterolaterally, unlike the narrow form of cRTr [70]. The inflation of this chamber renders the sagittal septum separating the posterior chambers incomplete, allowing a broad confluence between them (Fig 6D). Given this unusual inflation and the topographic similarity to both cRTr and MBd, the posterior chamber of *Siamraptor* appears to be formed by the fusion of chambers occupied by cRTr and MBd in other avetheropods.

#### Inner ear (endosseous labyrinth).

The cranial endocast also preserves the semicircular canals, cochleae, and vestibule of the inner ear. The inner ear measures approximately 112.8 mm dorsoventrally and 83.5 mm anteroposteriorly. The region outlined by semicircular canals is strongly triangular in lateral view (Fig 6A, B) as in *Carcharodontosaurus* [23] and *Giganotosaurus* [27]. The anterior and posterior semicircular canals are tall, slender and approximately triangular in posterolateral and anterolateral views, respectively. The anterior semicircular canal extends dorsally above the level of the posterior semicircular canal. The lateral semicircular canal is oval in dorsal view, with its main axis oriented anteroposteriorly.

## Discussion

### Identification as braincase and skull roof of *Siamraptor*

As described above, NRRU-F01020035 and F01020036 share an important feature supporting their affinity to Carcharodontosauria: the anteromedial corner of the supratemporal fossa excavating the frontal deeply, which is known as a possible synapomorphy of Carcharodontosauria or Carcharodontosauridae [43]. Furthermore, other carcharodontosaurian features can be observed in the part of NRRU-F01020035 that is not preserved in NRRU-F01020036: the supraoccipital knob substantially wider than the foramen magnum (an unambiguous synapomorphy of Allosauroidea [43]); fully split trigeminal foramen (a possible synapomorphy of Allosauroidea or Allosauria [43]); the internal carotid pneumatization developed as an opening (a possible synapomorphy of Allosauria [43]); tall nuchal crest (a possible synapomorphy of Carcharodontosauria [30,43] or late-diverging carcharodontosaurids [43]).

In addition to synapomorphies of the clades within Allosauroidea noted above, there are two features shared between in NRRU-F01020035 and F01020036 unique among allosauroids: the posterolaterally-directed frontoparietal suture on the skull table accompanied with the wedge-shaped anterior margin of the parietals; two deep pits on the lateral margin of the frontal at the anterior margin of the supratemporal fossa, despite their presence might be somewhat affected by the damage on the lateral margin of the frontal. These unique features indicate the closest affinity between NRRU-F01020035 and F01020036. In combination with the monospecific occurrence of Carcharodontosauria in the Ban Saphan Hin locality, both specimens can be identified as new materials of *Siamraptor suwati*. Nevertheless, given the current limitations of the fossil record, the presence of more than one closely related species of the same clade in the area cannot be entirely excluded.

### Phylogenetic analysis

**Fig 8:**
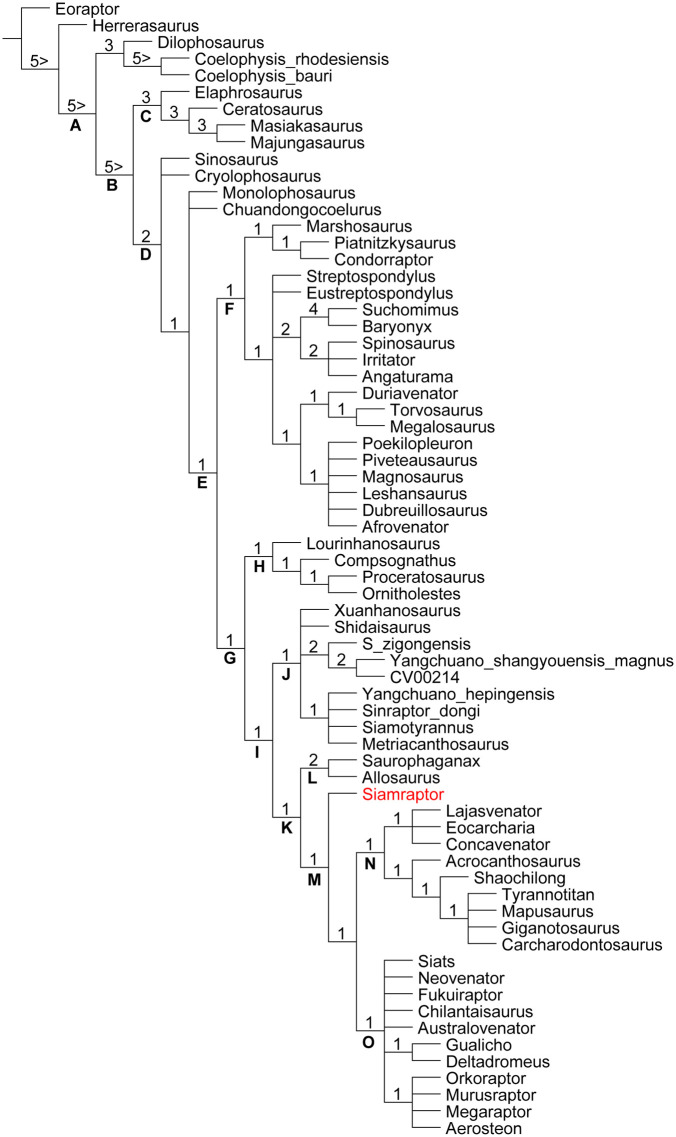
Strict consensus tree of theropods including *Siamraptor* with Bremer support values on each node. A, Neotheropoda; B, Averostra; C, Ceratosauria; D, Tetanurae; E, Orionidae; F, Megalosauroidea; G, Avetheropoda; H, Coelurosauria; I, Allosauroidea; J, Metriacanthosauridae; K, Allosauria; L, Allosauridae; M, Carcharodontosauria; N, Carcharodontosauridae; O, Neovenatoridae. Numbers at the top left of each node represent the Bremer support value.

The heuristic tree search using the data matrix composed of 367 characters (with character 201 inactivated following Coria and Currie [42]) distributed among 68 theropod taxa and incorporating the new materials described above in *Siamraptor* resulted in a total of 32,076 MPTs with 1,152 steps (CI = 0.370, RI = 0.661). The topology of the strict consensus tree of these MPTs is basically the same as that provided by Coria and Currie [42], except for the placement of *Siamraptor* at the basalmost position within Carcharodontosauria (Fig 8), which is consistent with the phylogenetic position suggested in the original description of *Siamraptor* [5]. Although the phylogenetic placement of megaraptorans remains debated and may vary depending on the dataset employed, they are conservatively treated here as members of Carcharodontosauria in accordance with the results of the present analysis. This treatment allows their inclusion as an important comparative taxon, while acknowledging that alternative affinities, such as with Tyrannosauroidea or Coelurosauria [19,32,33], cannot be entirely ruled out.

This analysis revealed that the braincase of *Siamraptor* exhibits the wide supraoccipital knob as an allosauroid synapomorphy [character 88 (state 1); reversed in *Murusraptor*], the opening of the internal carotid pneumatization as an allosaurian synapomorphy [ch. 107 (2); convergent with *Eustreptospondylus*] and the tall nuchal crest as a carcharodontosaurian synapomorphy [ch. 70 (1); convergent with *Majungasaurus*], in addition to known other synapomorphies seen in the remaining cranial and postcranial elements [5]. Furthermore, this analysis presented three diagnostic features for *Siamraptor* in the braincase: the shallow ventral extension of the subcondylar recess [ch. 101 (1); convergent with some ceratosaurs and some megalosauroids], the obtuse angle between the skull roof and the occiput in lateral view [ch. 103 (1); convergent with *Sinraptor* and the clade comprising *Shaochilong* and Carcharodontosaurinae] and the absence of the median ridge separating left and right exits of CN VI [ch. 105 (1); convergent with *Giganotosaurus* and *Carcharodontosaurus*].

### Implications for the braincase morphology of Allosauroidea

*Siamraptor* shares several characters with other carcharodontosaurs that are known to be possible synapomorphies of Carcharodontosauria, and some of these are also supported as synapomorphies in the present phylogenetic analysis. On the frontal, the anteromedial corner of the supratemporal fossa partially roofed by the pronounced dorsal rim [ch. 71 (1)] has been observed only in some late-diverging carcharodontosaurs, so that has been regarded as a possible synapomorphy of Carcharodontosauria or Carcharodontosauridae [43]. Although its absence in a late-diverging neovenatorid *Murusraptor* [31] may supports the latter hypothesis, its presence in *Siamraptor* rather supports the former hypothesis; a synapomorphy of Carcharodontosauria lost in Neovenatoridae. Lack of enough fossil records on neovenatorid frontals makes it difficult to when and how this character state was lost in this lineage, so that further discoveries on neovenatorid frontals are required to reveal this point. The function of this fossa has recently been hypothesized to house blood vessels that perform a thermoregulatory function to moderate eye and/or brain temperatures [71]. This suggests that at least some carcharodontosaurs may have had a specialized thermoregulatory function in this region compared to other theropods.

In the skull roof, both the frontals and the parietals are unfused in early-diverging allosauroids such as *Sinraptor* [10,11] and *Allosaurus* [13,15] as well as a neovenatorid *Murusraptor* [31], but are fused in carcharodontosaurids such as *Acrocanthosaurus* [15], *Carcharodontosaurus* [22], *Meraxes* [24] and *Giganotosaurus* [25]. Although the unfused conditions of some allosauroids may be due to their ontogenetic stages [10,32], at least parietals are usually fused during early ontogenetic stage in theropods [31,72], so that its presence or absence may have some phylogenetic implications, although this feature was not coded as a phylogenetic character in the present analysis. In this case, the median fusion of the skull roof is at first glance a synapomorphy of Carcharodontosauridae. However, the combination of fused parietals and unfused frontals in a non-carcharodontosaurid carcharodontosaur *Siamraptor* indicates that the fused parietals is a synapomorphy of Carcharodontosauria, and the median fusion was extended to the frontals in Carcharodontosauridae to make the skull roof more robust, whereas the fusion was reduced in Neovenatoridae.

The obtuse occipitofrontal angle [ch. 103 (1)] is widely distributed within allosauroid lineage as seen in *Sinraptor*, *Shaochilong, Carcharodontosaurus*, *Meraxes* and *Giganotosaurus*, but not in *Allosaurus* and *Acrocanthosaurus* [24,25,31,43]. In *Murusraptor*, although this angle has been regarded as obtuse [30], another research focused on its braincase revealed that the angle is rather acute [31] as scored in the present phylogenetic analysis. Because of such a distribution pattern, this character state has been interpreted as a synapomorphy of late-diverging carcharodontosaurids convergent with Metriacanthosauridae [43]. The presence of this character state in a basal carcharodontosaur *Siamraptor* increases the possibilities of two other scenarios as follows: (1) a synapomorphy of Carcharodontosauria convergent with Metriacanthosauridae but lost in *Acrocanthosaurus* and Neovenatoridae; (2) a synapomorphy of Allosauroidea independently lost in Allosauridae, *Acrocanthosaurus* and Neovenatoridae. However, the original hypothesis is more parsimonious than the two alternatives, as it requires fewer evolutionary changes, even if the obtuse occipitofrontal angle was independently acquired in *Siamraptor*. The obtuse occipitofrontal angle was regarded as an indicator of a horizontal craniocervical posture and a slightly upturned skull posture in *Ceratosaurus* [73]. Furthermore, the skull roof of *Siamraptor* forms the angle of about 15 degrees with the plane of lateral semicircular canal, indicating that the skull is slightly upturned when the lateral semicircular canal is parallel to the ground surface as in *Ceratosaurus* [73] but unlike carcharodontosaurids, in which the skull roof is oriented horizontally (Fig 1 in [74]) or slightly anteroventrally [65]. Although the orientation of lateral semicircular canal does not absolutely indicate the natural head posture [75], in combination with the obtuse occipitoparietal angle, the head of *Siamraptor* can be regarded as relatively upturned than other basal allosauroids.

The tall nuchal crest [ch. 70 (1)], which has been recognized as a possible synapomorphy of Carcharodontosauria [30,43], is recovered as an unambiguous synapomorphy in the present analysis due to its presence in the earliest-diverging carcharodontosaur, *Siamraptor*. Although a tall nuchal crest is also present in Abelisauridae, the contribution of the squamosal to the crest is dorsoventrally deep in that clade, whereas there is little or no such contribution in other theropods [58,76,77]. The present character (ch. 70) refers to the relative height of the nuchal crest and does not capture differences in its osteological composition. Therefore, a tall nuchal crest with little or no squamosal contribution is possibly a feature unique to Carcharodontosauria. Such nuchal crests can provide large areas and lever arms for cervical axial muscles supporting dorsiflexion at the craniocervical joint, possibly as a response to the greater mass of their heads [78–80].

The presence of fully split trigeminal foramina [ch. 104 (2)] has been regarded as a possible synapomorphy of Avetheropoda or Allosauroidea convergent with Megalosauria [43], as seen in allosauroids *Allosaurus* [15,16] and *Murusraptor* [30] as well as a megalosauroid *Piveteausaurus* [81]. However, the trigeminal foramina are merged into a single opening in *Acrocanthosaurus* as in most other theropods [18,28,31]. In addition, although the conditions of *Sinraptor* and *Giganotosaurus* have been scored as fully split [30,42–45], they have been reported as having partially split [10,56] and single [25] openings, respectively, and were scored accordingly in the present phylogenetic analysis. Similarly, despite *Carcharodontosaurus* having been regarded as fully split [30,42–45] as seen in *C. iguidensis* [21], only a single opening has been reported in *C. saharicus* [21,23] so that this genus was scored as having both states in the present analysis. Furthermore, although the scoring of *Shaochilong* for this character has remained unknown in previsou studies [30,42–45], the presence of a single trigeminal foramen has been reported [28], and this taxon was therefore scored as exhibiting the primitive condition in the present study. The differences in reported conditions discussed above primarily reflect revisions of previous scorings based on re-examination of the literature, rather than direct evidence for biological or infraspecific variation. The presence of fully split trigeminal foramina in *Siamraptor* in combination with the distribution pattern revised above suggests that the fully split trigeminal foramina may represent a synapomorphy of Allosauria, while also indicating a complex evolutionary history within the group.

When the frontal is horizontal, the positions of the trigeminal foramina are anterior to the level of the nuchal crest in most theropods including *Sinraptor* and *Allosaurus* [25]. The position becomes more posterior in carcharodontosaurs to some degree, at least at the level of the crest as in *Carcharodontosaurus iguidensis* and *Acrocanthosaurus* [21], or posterior to the level as in *Carcharodontosaurus saharicus* and *Giganotosaurus* [25]. The trigeminal foramina of *Siamraptor*, with the one located at the level and the others located posterior to the level, represent an intermediate condition among these carcharodontosaurs.

The exit of CN VII splits into two in *Acrocanthosaurus* and possibly in *Giganotosaurus* [17,18] and thus it has been regarded as a character indicating carcharodontosaurid affinity [28]. Therefore, the absence of this character in *Siamraptor* supports its phylogenetic position as not a carcharodontosaurid. However, the presence of the partially-splitting condition represented by an hourglass-shaped opening in a neovenatorid *Murusraptor* [31] indicates that at least the intermediate condition was acquired immediately before the divergence of Carcharodontosauridae and Neovenatoridae, probably after the divergence of *Siamraptor*.

The exits of CNs X–XII are described as present lateroventral to the occipital condyle in the original phylogenetic dataset, whether the condition is primitive or derived [30,42–45]. However, these exits are usually situated anterolateral to the occipital condyle and lateroventral to the foramen magnum in both theropods with the primitive (e.g., [10,14,58,73,82–84]) and derived (e.g., [18,85–88]) conditions. Therefore, the description of this character state is revised in the present study. Although the presence of the three foramina for CNs X–XII has been reported in *Shaochilong* [28] and *Giganotosaurus* [64], they had been scored as having only two foramina [30,42–45]. Considering the lack of justifications about these conflicts in the latter studies [30,42–45], these two taxa are regarded as having three foramina for CNs X–XII in the present study. Similarly, although *Carcharodontosaurus* has been scored as unknown for this character [30,42–45], the presence of three foramina was reported previously [21], so that has been re-coded in the present study. *Acrocanthosaurus* has three foramina within the paracondylar pocket on the left side while having only two on the right side [18]. These three were identified as CNs IX, X + XI and XII [18], so that there are only two exits for CNs X–XII contrary to the derived condition retained in the matrices used in the previous studies [30,42–45]. However, it should be noted the combination of a single foramen for CNs IX–XI, which is present on the right side, and two foramina for CN XII is also likely to be, as the latter are usually seen in derived theropods [28] including most allosaurs such as *Sinraptor* [56], *Shaochilong* [28], *Carcharodontosaurus* [23], *Giganotosaurus* [27] and *Murusraptor* [31]. In addition, if CN IX has a separate exit, it may open around the columellar recess anterior to the metotic strut as in *Carcharodontosaurus* [23], *Murusraptor* [31] and *Siamraptor* (Fig 4B). Therefore, given the uncertainty in identifying the exact nerve assignments of these foramina, the possibility that the third foramen on the left side of *Acrocanthosaurus* represents an additional foramen of CN XII cannot be excluded. Accordingly, this taxon was treated as potentially exhibiting both the primitive and derived conditions in the present analysis. As a result, in combination with the presence of the three foramina in *Siamraptor*, the derived character state is revealed as an unambiguous synapomorphy of Carcharodontosauria convergent with Megalosauroidea, so that the intermediate condition of *Acrocanthosaurus* might be an autapomorphic feature of this taxon.

The caudal tympanic recess seen in *Siamraptor* is also known in *Acrocanthosaurus* and coelurosaurs, but has been reported as absent in a basal allosauroid *Allosaurus* [14], later-diverging carcharodontosaurids *Giganotosaurus* and *Carcharodontosaurus* [21,70], and a later-diverging neovenatorid *Murusraptor* [31]. Although the true absence of such a recess can be difficult to assess [89], the presence of a distinct caudal tympanic recess remains important in the present context. If the presence of this recess represents a plesiomorphic condition of Allosauroidea, as suggested by Dufeau [70], it appears to have been retained in Carcharodontosauria but secondarily lost in later-diverging taxa of Carcharodontosauridae and Neovenatoridae.

The fusion of the chambers for the caudal diverticulum of the rostral tympanic recess (cRTr) and the medial basioccipital diverticulum (MBd) seen in *Siamraptor* is also known in ornithomimosaurs, oviraptorosaurs, and therizinosaurs, but is unknown in more basal theropods [70]. Therefore, it can be considered an autapomorphy of *Siamraptor* among allosauroids, but it is worth considering that it could be a plesiomorphy or synapomorphy of Carcharodontosauria, as it gives some idea to understand the unique condition of *Murusraptor*. In *Murusraptor*, the position of cRTr and MBd is occupied by the “basisphenoidal recess”, which has a posteriorly elongated shape as in cRTr and a paired opening at the dorsal apex of the basisphenoid recess as in MBd [31,70]. In the latter scenario, the caudal chamber is originally occupied only by cRTr in Allosauroidea, but occupation by MBd was initiated at the latest in Carcharodontosauria and then completed in Neovenatoridae. For *Murusraptor*, this scenario is consistent with the presence of only one pair of posteriorly elongated chambers connected only to the basisphenoid recess, but is inconsistent with the lower degree of inflation.

## Conclusions

The braincases of *Siamraptor* recovered in the present study provided unknown aspect of its cranial anatomy, including two diagnostic features, many similarities and several differences with other allosauroids, and many characters enriching the data matrix of the phylogenetic analysis confirming its phylogenetic position as an earliest-branching carcharodontosaur. Detailed comparisons with other allosauroid braincases revealed several synapomorphies of Carcharodontosauria, some of which are inherited by carcharodontosaurids but not by neovenatorids. Some characters observed in the braincases also have functional implications, such as the presence of the specialized thermoregulatory system and adaptations for supporting large head that are shared with carcharodontosaurids. In addition, somewhat upturned head posture was indicated for *Siamraptor* by the angles of the occiput and the lateral semicircular canal relative to the skull roof. Because the earliest-branching carcharodontosaur *Siamraptor* is still known from limited elements, future works in Nakhon Ratchasima will provide further information on this taxon and recover unknown aspects of the earliest stage of carcharodontosaurian evolution.

## Supporting information

S1 File Data matrix used in the phylogenetic analysis in TNT format. (TNT)
